# Bringing Community Ecology to Bear on the Issue of Antimicrobial Resistance

**DOI:** 10.3389/fmicb.2019.02626

**Published:** 2019-11-15

**Authors:** Aabir Banerji, Michael Jahne, Michael Herrmann, Nichole Brinkman, Scott Keely

**Affiliations:** Office of Research and Development, Center for Environmental Measurement and Modeling, US Environmental Protection Agency, Cincinnati, OH, United States

**Keywords:** antibiotic, biofilm, competition, consortia, mutualism, predation, indirect effects

## Abstract

Antimicrobial resistance (AMR) is a global concern, pertaining not only to human health but also to the health of industry and the environment. AMR research has traditionally focused on genetic exchange mechanisms and abiotic environmental constraints, leaving important aspects of microbial ecology unresolved. The genetic and ecological aspects of AMR, however, not only contribute separately to the problem but also are interrelated. For example, mutualistic associations among microbes such as biofilms can both serve as a barrier to antibiotic penetration and a breeding ground for horizontal exchange of antimicrobial resistance genes (ARGs). In this review, we elucidate how species interactions promote and impede the establishment, maintenance, and spread of ARGs and indicate how management initiatives might benefit from leveraging the principles and tools of community ecology to better understand and manipulate the processes underlying AMR.

## Introduction

**Figure 1:**
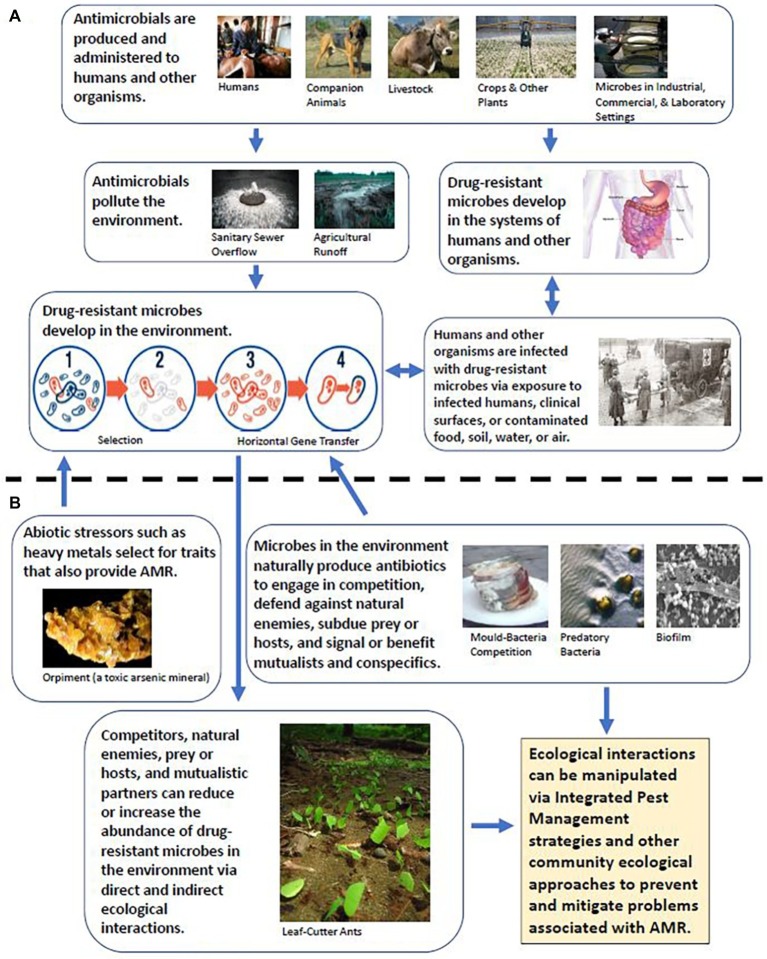
**(A)** Traditional view of AMR, wherein the environment and wildlife mostly represent opportunities for exposure and/or reservoirs of drug-resistant microorganisms. **(B)** Expansion of the traditional view of AMR to include the effects and potential management implications of species interactions.

As pathogens and other microbes become increasingly and more frequently resistant to antibiotics, concern is growing world-wide that the use of antibiotics for treating and preventing diseases in humans, animals, and plants is rapidly becoming less effective and unsustainable (Nerlich and James, 2009; Defoirdt et al., 2011; Stockwell and Duffy, 2012; Wellington et al., 2013; Aćimović et al., 2015). Antimicrobial resistance (AMR) may, in addition, threaten the health of ecosystems (Grenni et al., 2018) and the performance of businesses that rely on large-scale maintenance of microbial monocultures or AMR-related reporter genes for culinary and industrial fermentation processes (Teuber et al., 1999; Bourdichon et al., 2012; Shaw et al., 2016), food and nutritional supplement cultivation (Richmond and Preiss, 1980; Hallmann and Rappel, 1999; Mišurcová et al., 2012; Wells et al., 2017), bioremediation (Jebelli et al., 2018), energy harvesting and biofuel production (Arora et al., 2015; Wang et al., 2015; Oliver et al., 2016), and the derivation of bioprospects (Strobel and Daisy, 2003; Ferrer et al., 2016) such as dyes (Tuli et al., 2015; Sen et al., 2019) and self-healing concrete (Seifan et al., 2016). Although many strategies to mitigate the threat and current impacts of AMR are presently being explored and enacted, such as altering how antibiotics are administered and regulated to sustain or improve their effectiveness (Fridkin and Gaynes, 1999; Drusano, 2003; Pruden et al., 2013; Chakradhar, 2016), discovering or developing new classes of natural and synthetic antibiotics to supplement or replace the old (Livermore, 2011; Moloney, 2016; Wiese and Imhoff, 2019), and employing bacteriocins and other scalable alternatives to the use of antibiotics (Joerger, 2003; Reardon, 2015; Czaplewski et al., 2016; Willing et al., 2018), the problem remains. At the root of this problem is the One Health nature of AMR; i.e., the interconnectedness among human, animal, and environmental systems (Figure 1A; Collignon and McEwen, 2019).

To begin to address this interconnectedness, we must acknowledge that resistance to antibiotics may not be the primary or original (evolutionary) purpose of antimicrobial resistance genes (ARGs). ARGs occur naturally in the environment, where they have been shown or posited to confer protection against toxins such as heavy metals and host-produced biocides and to perform important roles in cellular processes such as quorum sensing (the detection of conspecific or cooperative cells in the environment, associated with cell-to-cell communication/signaling and group activity) and biosynthesis (the enzymatic conversion of simple compounds into more complex products by living things; Figure 1B; Allen et al., 2010; Martinez, 2018). Indeed, ARGs are detected even in habitats that have historically been sheltered from antibiotic pollution and other forms of anthropogenic disturbance (McArthur et al., 2016; Van Goethem et al., 2018). Many antibiotic-producing microbes possess ARGs, ensuring that they are resistant or immune to their own antibiotics (Benveniste and Davies, 1973). Antibiotic-producing microbes may thus be a source of ARGs in sympatric species *via* horizontal gene transfer (Jiang et al., 2017; Ringel et al., 2017) or select for AMR in the targets of their antibiotics (Wellington et al., 2013), resulting in an “arms race” of cyclical coevolutionary dynamics known as “Red Queen” dynamics (Baron et al., 2018; Decaestecker and King, 2019). From the standpoint of managing AMR, this poses two separate challenges. First, for any new class of antibiotic discovered in nature, there may already be a corresponding suite of ARGs among or transferrable to pathogens (Bengtsson-Palme and Larsson, 2015). Second, efforts to monitor changes in ARG prevalence and identify areas of concern based on comparison to a “least disturbed” reference site may need to account for natural environmental variations in the reference site that favor the natural producers or production of antibiotics. Reference sites may themselves become areas of concern under certain conditions and should, in any case, be critically reviewed to ensure their appropriateness as a baseline of comparison (White and Walker, 1997; Whittier et al., 2007; Berendonk et al., 2015; Rothrock et al., 2016; Vikesland et al., 2017).

High-throughput “-omics” techniques, including genomics, transcriptomics, metabolomics, and proteomics, have greatly enhanced our ability to detect and quantify presence of antibiotic-resistant strains, identify modes of transmission of ARGs, and illuminate complex expression pathways and epigenetic mechanisms (Cockerill, 1999; Cohen et al., 2015; Huijbers et al., 2015; Motta et al., 2015; dos Santos et al., 2016; Anjum et al., 2017). These techniques can be useful for establishing best management practices to reduce antimicrobial resistance (Durante-Mangoni and Zarrilli, 2011; Cohen et al., 2015), estimating and monitoring risk of exposure to antibiotic-resistant pathogens (Brul et al., 2012; Tyson et al., 2015; Haddad et al., 2018; Brockhurst et al., 2019), and assessing environmental impacts of antibiotic pollution (Cairns et al., 2018b; Danner et al., 2019). They have helped to uncover critical roles that the environment plays in determining the establishment, maintenance, and spread of ARGs *via* mechanisms such as co-selection, co-resistance, cross-resistance, hypermutation, and exchanges of plasmids, transposons, and integrons (Seiler and Berendonk, 2012; Singer et al., 2016; Pal et al., 2017). However, while the value of these insights and of the as-yet untapped potential of -omics techniques in general cannot be overstated, elucidation of the molecular biology of AMR should complement, not overshadow, our understanding of underlying ecological processes, such as the relationships that microbes have with other species in the environment (US Centers for Disease Control and Prevention and UK Science and Innovation Network, 2018; Brockhurst et al., 2019). Here, we review some of these ecological relationships, discuss their implications for AMR management, and highlight existing frameworks in community ecology that could be used to address the problem of AMR holistically and robustly.

## Species Interactions Involving Antimicrobial Resistance

### Competition

Although the intracellular and ecological functions of antibiotics in nature are varied and, in many cases, uncertain, at least some of the organisms that produce antibiotics appear to use them for allelopathy, “chemical warfare” with competing species (Sturz et al., 1998; Baquero et al., 2009; Raaijmakers and Mazzola, 2012; Chevrette and Currie, 2019). *Penicillium notatum*, for example, the mold famously discovered by Alexander Fleming to produce penicillin, was found, in that instance, to compete for resources and space with the bacterium *Staphylococcus aureus* (Demain and Elander, 1999; Bennett and Chung, 2001). This situation could increase the prevalence of ARGs in the environment, as ARG-possessing antibiotic producers outcompete their targets or as targets evolve ARGs in response to their competitors’ antibiotics. Even if microbes do not engage in allelopathy, possessing ARGs may increase their biological fitness due to co-benefits of the resistance mechanisms (e.g., ARGs coding for or regulating efflux pumps simultaneously provide resistance to heavy metals and other toxic compounds; Allen et al., 2010). On the other hand, if resource limitation is the predominant driver of microbial population dynamics and phenotypic plasticity in the expression of ARGs is insufficient to reduce costs (Auld et al., 2009), then microbes that do not house ARGs may have a competitive advantage over microbes that do, due to the energetic expenses or other physiological tradeoffs associated with ARGs (Kang and Park, 2010; Basra et al., 2018).

Moreover, under certain conditions, ARG-possessing microbes might facilitate rather than competitively exclude other microbes (Klümper et al., 2019). This has been shown to occur in one of two ways: through mutualism, which we describe in the next section, or through exploitation of “leaky” or “public” resistance mechanisms. For example, susceptible microbes can benefit from neighboring ARG-possessing competitors’ production and release of signaling compounds such as indole, a compound which activates drug efflux pumps and oxidative-stress protective mechanisms (Lee et al., 2010). As previously mentioned, antibiotics themselves may serve as signaling compounds, though they are more likely to be utilized by conspecifics and mutualistic symbionts than by competitors (Allen et al., 2010). Relative costliness of ARGs may create a selection pressure favoring exploiters over resistance builders, so that only the microbes that require ARGs for essential life processes retain their ARGs over time. Such “race to the bottom” coevolutionary dynamics are known as “Black Queen” dynamics (Morris et al., 2012; Cairns et al., 2018a). Microbes exhibiting Black Queen dynamics would represent a best-case scenario for human priorities regarding the evolution of AMR in pathogens, since it would entail selection against ARGs even under conditions favoring AMR.

### Mutualism

The term “mutualism” refers to a relationship of mutually beneficial exchanges between different species (Hoeksema and Bruna, 2000). Certain mutualisms can enable microbes to acquire AMR without necessarily possessing their own independent ARGs. Two such mutualisms that have garnered attention in clinical settings and nutritional science are microbial biofilms and syntrophic consortia. Biofilms are structured associations of microbes that form on surfaces, including within food processing and water treatment facilities, various medical devices, and the human body (Donlan, 2002; Chmielewski and Frank, 2006; Ling et al., 2015; Kovach et al., 2017; Hu et al., 2018). They provide microbes with defenses against adverse conditions, increased efficiency in sequestering and assimilating nutrients, and other cooperative benefits (Jefferson, 2004; Nadell et al., 2016; Zhang et al., 2018). Syntrophic consortia are symbiotic associations of two or more microbial groups that allow for synthesis or degredation of substances that few or none of the constituent microbes would be able to synthesize or degrade on their own (Madigan et al., 2009; Morris et al., 2013; Bradáčová et al., 2019). Note that these characterizations are not mutually exclusive of one another: a biofilm can be a consortium and vice versa, depending on its location, form, and benefits.

The formation of biofilms can not only enhance the horizontal exchange of ARGs among constituent microbes (Molin and Tolker-Nielsen, 2003) but also confer AMR based on the physical and chemical structure of exopolysaccharides and other features of biofilm architecture shielding the constituent microbes’ cell envelopes (Mah and O’Tool, 2001; Jałowiecki et al., 2018). In addition, biofilms can provide resistance based on less intuitive mechanisms, such as slowed growth and inhibition of targeted metabolic processes (Olsen, 2015), achieved through the release of toxins by core constituent microbes (Lewis, 2005). Similarly, syntrophic consortia can allow microbes to gain AMR by creating enzymes in assembly-line fashion that degrade or inhibit antibiotics (e.g., β-lactamases; Olsen, 2015). This can be based on a small number of constituent microbes synthesizing the enzymes and the rest either supplying essential resources (Fan and He, 2011; Liu et al., 2017) or ameliorating factors such oxidative stress (Shatalin et al., 2011), or it can be based on multiple constituent microbes each synthesizing complementary parts of the enzymes (Burmølle et al., 2006; Islas-Espinoza et al., 2012). Mutualisms giving rise to exogenous or emergent AMR may call for the development of new methods of assessing antibiotic susceptibility that go beyond screening for conventional ARGs, at least in the case of the microbes known to engage in such mutualisms.

### Predation and Parasitism

Microbes are often the prey or hosts of other species, including other microbes. Use of these natural enemies as biological control agents to combat clinical pathogens is a hot-topic area of research (which we delve deeper into in a later section) and has been shown to be effective under certain circumstances (Kutateladze and Adamia, 2010), including where pathogens already exhibit AMR (Willis et al., 2016). In natural environmental contexts, the influence of predation and parasitism on ARGs and AMR is complicated by the fact that certain predators and parasites produce antibiotics to subdue their prey or hosts and certain prey and hosts produce antibiotics to defend themselves from predators and parasites. For instance, predatory myxobacteria utilize antibiotics (myxovirescin and corallopyronin) to subdue prey such as *Escherichia coli* (Xiao et al., 2011), as does the non-obligate predator *Aristabacter necator* Strain 679-2 (pyrrolnitrin, maculosin, and banegasine; Cain et al., 2003). Antarctic sponges of the genus *Crella* produce antibiotic steroids (norselic acids A–E) that deter predators such as the amphipod *Gondogeneia antarctica*, as well as protozoan parasites of the genus *Leishmania* (Ma et al., 2009). Antibiotic-producing consumers and resources, like other antibiotic-producing organisms, may be sources of ARGs in sympatric species and select for AMR in the targets of their antibiotics.

### Complex Associations and Indirect Effects

Although the broad categories of competition, mutualism, and predation/parasitism accommodate the entire spectrum of fundamental pairwise relationships within ecology (from mutually detrimental to mutually beneficial), these pairwise relationships do not exist in a vacuum. In the context of real-world ecological communities, mutualisms can enable predators and parasites to subdue their prey or hosts (Hwang et al., 1989; Mlot, 1997; Jones and Nishiguchi, 2004; Shiga, 2005), enable prey or hosts to fend off their predators and parasites (Soler et al., 2010; Pauli et al., 2014; Flórez et al., 2015; Van Arnam et al., 2018; Chevrette and Currie, 2019), and enable competitors to exclude their rivals (Preer et al., 1953; Brown et al., 2008; Mangla et al., 2008). Some of these complex associations and indirect interactions are mediated by antibiotics and ARGs. For example, the medicinal plant *Leptospermum scoparium* relies on endophytic bacteria to produce antibiotics such as phenazine and 2,4-diacetylphloroglucinol, which inhibit infection of the plant by pathogens such as *Pseudomonas syringae* pv. actinidiae (Wicaksono et al., 2018). Wicaksono et al. (2018) found that these bacteria were transmissible to other plants and could therefore be used for biological control of the plant diseases. Similarly, the pest beetle *Lagria villosa* relies on *Burkholderia gladioli* to produce icosalide, a lipocyclopeptide antibiotic that protects the beetle’s offspring from entomopathogenic bacteria (Dose et al., 2018), bacteria which predatory nematodes often rely on to envenomate prey (Mlot, 1997). Fungus-growing attine ants weed out microfungal parasites (competitors for the ants’ food) of the genus *Escovopsis* from their fungal gardens using antibiotics produced by a streptomycete bacterium that resides within their cuticles (Currie et al., 2003; Little and Currie, 2007). There is evidence to suggest that the ants might deliberately (arguably “artificially”) select their antibiotic-producing bacteria (Barke et al., 2011) and that the nature of the relationship has permitted the antibiotics to remain effective in controlling the parasitic fungi despite millions of years of coevolution (Pathak et al., 2019). In a study that had implications for agriculture, Li and Alexander (1988) found that use of antibiotic-producing soil inoculants can enhance crop yield and resilience by increasing colonization and nodulation by rhizobia.

Antibiotics and ARGs associated with symbionts of macroscopic hosts seem, at first glance, unlikely to be an important source of antibiotics and ARGs in clinical, industrial, or water treatment settings. If they have shared a long co-evolutionary history with their macroscopic hosts, the symbionts will likely have lost certain functional traits that are important to the fitness of their free-living counterparts (Bennett and Moran, 2015) and have as restricted a geographical distribution as their hosts (Martiny et al., 2006; Joseph et al., 2016). Three caveats to consider, however, are that: (1) even if the symbionts die when their hosts die, the symbionts’ antibiotics and genes are released into the environment; (2) animal migration, human globalization, and other modes of dispersal all permit spread to management-relevant environments (Brown and Barker, 1999; Molmeret et al., 2005; Allen et al., 2010; Forsberg et al., 2012; Zurek and Ghosh, 2014); and (3) many hosts of microbial symbionts are known vectors of disease. Investigating what Galimand et al. (2006) have referred to as a “clinically ominous event,” Hinnebusch et al. (2002) demonstrated that horizontal gene transfer among the microbes inside the Oriental rat flea *Xenopsylla cheopis* may have been the cause of AMR in *Yersinia pestis* strains isolated from bubonic plague patients in Madagascar.

## Community Ecological Approaches to Addressing Antimicrobial Resistance

While using methods such as -omics to comprehensively profile microbial communities both in and out of clinical settings is still a major priority, the biological data produced through these methods must ultimately be interpreted and synthesized for there to be progress in addressing the problem. The frameworks for this synthesis currently exist within community ecology. Two that are well-established and appropriate for the task are “integrated pest management” and “ecological succession.”

### Integrated Pest Management

Integrated pest management (IPM) is a strategy designed to safeguard human health and the environment while avoiding, attenuating, or delaying pest outbreaks or associated damages (Kogan, 1998; Liang et al., 2015). Historically, the major focus of IPM has been minimization of pesticide use and enhancement of its efficacy in targeting herbivorous insects, weeds, and vectors of disease. Part of the motivation for this focus is that pesticides, globally, have promoted pesticide resistance among target species, adversely impacted non-target and beneficial organisms, and, in some cases, proven to be persistent in the environment (Barzman et al., 2015). The issue is analogous to that of antibiotics promoting AMR and other adverse outcomes. Although Magarey et al. (2019) and others (Fidler, 1998; Orzech and Nichter, 2008) have noted that complex cultural, legal, and socioeconomic factors underlie both issues and must be addressed for management practices to be successful, we focus here on providing basic understanding of relevant IPM approaches and elucidating how they might be applied in the context of AMR. Specifically, we consider the approaches of biological control and habitat manipulation.

Biological control is the use of living organisms to control pests. As previously stated, microbes, including those possessing ARGs, have natural enemies such as predators and parasites. A few studies highlight the potential of using these natural enemies to control pathogens responsible for post-harvest diseases of fruits and vegetables (Wilson et al., 1993), tree and woody plant diseases (Cazorla and Mercado-Blanco, 2016), and even diseases within humans and domesticated animals (Negus et al., 2017). Among the presently favored candidate biological control agents are protozoan bacterivores and plasmid-dependent bacteriophages. By decreasing microbial population abundance (the prerequisite for heritable variation and population viability) and exacting opposing selection pressures (compared to those exacted by antibiotics, other natural enemies, or competing microbes), these consumers can theoretically replace antibiotics or enhance the effectiveness of antibiotics and reduce the likelihood of AMR (Hiltunen et al., 2017; Cairns et al., 2018a,b).

Unfortunately, evidence is conflicting as to whether there are tradeoffs between AMR and defense against natural enemies. For example, whereas Chen et al. (2017b) found that AMR coincides with increased susceptibility to bacteriophages, Allen et al. (2017) found that AMR is associated with increased resistance to bacteriophages. If indeed AMR provides the co-benefit of defense against natural enemies, introduction of natural enemies may amplify the effectiveness and prevalence of AMR rather than decrease it. Moreover, in the environment of a gut or epiphytic microbiome, bacteriophages or other natural enemies of microbes may affect microbes that are necessary or beneficial for overall health more than they affect ARG-possessing pathogens. Equivalently, the natural enemies may prevent competitively inferior ARG-possessing microbes from being excluded by other microbes (Bohannan et al., 2002), in a manner akin to “keystone predation” (where apex predators preferentially feed on superior resource competitors, promoting biodiversity among the prey; Amarasekare, 2008).

Due to these concerns, biological control of pathogens, especially within humans and domesticated animals, requires forethought and the means to ensure that “ecological release” of the biological control agents and other potential adverse outcomes do not occur within or on the patients (Miller and Aplet, 1993; Louda et al., 2003; Shanmuganathan et al., 2009). Barratt et al. (2010) noted that numerous recent advances in risk assessment methodologies have helped to minimize the possibility of adverse outcomes of biological control. These include multi-factorial assessments of control agent viability, quarantine laboratory host range testing, pre- and post-release studies incorporating mechanistic population dynamics models, and meta-analysis of cumulative data from past introductions. Each of these advances (with the possible exception of meta-analysis of past introductions) can be tailored to evaluate the efficacy of biological control of pathogens and AMR. For example, comprehensive profiles of microbial communities both in and out of clinical settings can be assembled *via* sampling and manipulative experiments and be used to establish the host/prey specificity of putative biological control agents or select the most appropriate stage of infection or population growth at which to introduce biological control agents.

Habitat manipulation entails altering the physical, chemical, or biological structure of the landscape to inhibit pests or to benefit or manipulate the natural enemies and competitors of the pests. This includes creating or manipulating breeding grounds and refuges against adverse abiotic conditions, such as overwintering sites in terrestrial systems (Griffiths et al., 2008) or zones of oxygenated hypolimnion in aquatic systems (Klumb et al., 2004). It also includes providing incentives for pests to leave or reduce their impacts (e.g., trap crops; Badenes-Perez et al., 2004), targeting the pests’ symbionts and vectors (Beard et al., 2002; Pocquet et al., 2014), and introducing alternative prey or hosts to amplify the effects of natural enemies *via* apparent competition (Holt and Bonsall, 2017). In the context of managing AMR, the “landscape” may be outdoor environments and open public spaces serving as source pools of ARGs or hubs of transmission; indoor facilities and equipment; or individual vector, host, or patient microbiomes. At the larger scale, habitat manipulation might thus entail familiar tactics such as regulating and remediating pollutants, employing safe and sustainable hygiene practices, and removing conditions that favor human, plant, or animal exposure to vectors of disease. In the case of microbiomes, analogues of altering abiotic conditions, targeting of pests’ symbionts, and introducing alternative prey or hosts to amplify natural enemies’ effects might include manipulating dietary nutrients, utilizing prophylactics and probiotics (see, however, Gueimonde et al., 2013 for a cautionary perspective on this approach), and targeting non-pathogenic (or less pathogenic) mutualistic partners of pathogens rather than the pathogens themselves. Recent preliminary studies have even suggested that microbes not ordinarily present in the hosts’ organs could be employed for this purpose; e.g., photosynthetic cyanobacteria could be injected into the heart to convert blood carbon dioxide to oxygen and thereby inhibit the causes and symptoms of heart disease and necrosis (Cohen et al., 2017). Treatment strategies that counteract the benefit of exogenous or emergent AMR, such as the use of agents that disrupt quorum sensing or target specific components of the biofilm matrix and the use of biofilm-penetrating antibiotics, would also be a worthwhile extension of the principle of habitat manipulation (Donlan, 2000; Sully et al., 2014).

### Ecological Succession

Ecological succession is the phenomenon of biological communities forming or re-assembling and changing over time following natural or anthropogenic disturbances. These disturbances can include influx or loss of resources or habitat structure (e.g., due to urban development), invasion of the ecological community by exotic species, and changes in abiotic conditions due to seasonality or pollution. They can also often be autochthonous, as in the case of pioneer lichen creating soil from bare rock (Shure and Ragsdale, 1977). Patterns of succession affect functional traits within species and interaction strengths among species and depend on factors such as disturbance severity and species’ dispersal limitations (Chang and Turner, 2019). Ecological succession theory has been developed within classical microbiology, even including versions of the concepts of *r*- and *K*-strategists (Dorodnikov et al., 2009). However, two popular microbiological paradigms have created some divergence between microbial ecological succession theory and classical ecological succession theory: that of “everything is everywhere, and the environment selects” (O’Malley, 2007) and of microbial utilization of resources being determined thermodynamically by reduction-oxidation reaction-related processes (a.k.a. the “Redox Tower”; Delattre et al., 2019). Through the lens of these paradigms, antibiotics constitute two separate forms of disturbance: a selection pressure against non-resistant microbes (which would release some of their metabolic resources and space, making them available to other microbes) and a metabolic resource for AMR-exhibiting microbes. Combining this concept with an understanding of the previously mentioned syntrophic consortia, it may be possible to predict the rate at which and extent to which antibiotics would be enzymatically degraded in the presence of particular microbial assemblages and the likelihood of ARG spread within those assemblages. This information could be used to assess or predict environmental impacts and legacy effects of antibiotics or to develop novel mitigation strategies. To some extent, it has already been used to develop or enhance certain bioremediation techniques (US Environmental Protection Agency, 2013).

However, real-world microbial succession is typically more complicated (Chen et al., 2017a). Within microbiomes of macroscopic hosts, various potential metabolic resources can become available simultaneously (e.g., *via* mixed diets), and host immune responses, natural enemies, and competitors of various kinds may also be present. Furthermore, as stated previously, microbes can obtain exogenous or emergent AMR *via* exploitation of ARG-possessing microbes, potentially promoting Black Queen coevolutionary dynamics (see *Competition* subsection of Species Interactions Involving AMR). Depending on the level of impact it has on the ARG-possessing microbes, this could allow for coexistence or be analogous to successional replacement of “pioneer” and “nurse” plants (Whittaker, 1993; Tewksbury and Lloyd, 2001). Conceptual models within classical ecological succession theory that could be used to predict such outcomes include that of “alternative stable states” (Fukami and Nakajima, 2011) and that of the “Stress-Gradient Hypothesis” (Bertness and Callaway, 1994). The latter, originally developed for terrestrial plant communities (and later refined by Malkinson and Tielbörger, 2010), posits that, among potential competitors, intense grazing pressure or abiotic stress can increase the prevalence and importance of facilitative interactions such as “associational defense” and “neighborhood habitat amelioration,” provided that the competitors possess adequate complementarity in their physiological traits. Studies of AMR across gradients of antibiotic pollution, other abiotic stress, and bacterivore biodiversity (e.g., Baquero and Negri, 1997; Dunivin and Shade, 2018; Harmand et al., 2018) could be used to parameterize numerical simulations based on the hypothesis and test whether facilitative interactions in the form of AMR-conferring multi-species syntrophic consortia and biofilms occur as predicted along these gradients.

## Discussion

AMR is a global human health concern, exacerbated by human population growth and socioeconomic inequity (Allcock et al., 2017; Jensen et al., 2019), climate change (MacFadden et al., 2018), and biological warfare/terrorism (Inglesby et al., 2000). It is also a pressing concern within the realms of industry, food production, and environmental health (Figure 1). The approach of “kill everything susceptible for as long as it is susceptible” has proven to be short-sighted and costly, with far-reaching implications. The relationships that microbes have with other species are an integral component of AMR ecology and should therefore be accounted for in both management initiatives and basic research. As we have described above, these relationships can have significant and sometimes multifaceted roles in determining the prevalence and distribution of AMR and ARGs. They and their effects call for subtle changes in our thinking regarding the boundaries of the problem that allow for innovations in the management of AMR, innovations that could easily and appropriately draw from existing frameworks within the field of community ecology. Priorities that would enable this progress include further characterizing AMR ecology in complex microbial communities and formally articulating and evaluating the links between this ecology and the risks to human and environmental health *via* mechanistic models and experimental tests of ecological theory.

Among the many questions still needing to be addressed regarding AMR ecology is what impact ARGs have on the longevity and infectivity of pathogens in the environment, outside of their hosts. Although many studies have traced ARGs introduced through human activities such as the generation of wastewaters, few have examined how the microbes associated with these ARGs persist and interact with other species in their new surroundings (Rodriguez-Mozaz et al., 2015; Bengtsson-Palme et al., 2017; Smalla et al., 2018). Knowing how frequently horizontal gene transfer of ARGs occurs among mutualistic symbionts, across trophic levels of microbial food webs, and in general (and, more importantly, knowing why) would enable researchers to better estimate the probability of non-pathogenic organisms with ARGs spreading resistance to the pathogens of humans, plants, and domesticated animals (Cairns et al., 2018a,b). Ideally, future research and mitigation efforts will leverage the fundamentals of modern microbial ecology, bringing together the principles and tools of molecular genetics, classical microbiology, and community ecology to allow us to better understand and manage the processes driving this complex challenge.

## Author Contributions

AB provided the premise and framework of the manuscript and contributed the bulk of the writing and literature review pertaining to ecological theory and applications. MJ, MH, NB, and SK each contributed to the background regarding molecular/genetic aspects of AMR and AMR-related exposure risks, assisted in proof-reading, and helped to finalize the overall scope of the manuscript.

### Conflict of Interest

The authors declare that the research was conducted in the absence of any commercial or financial relationships that could be construed as a potential conflict of interest.
